# Mineral and bone disorder management in hemodialysis patients: comparing PTH control practices in Japan with Europe and North America: the Dialysis Outcomes and Practice Patterns Study (DOPPS)

**DOI:** 10.1186/s12882-018-1056-5

**Published:** 2018-10-05

**Authors:** Suguru Yamamoto, Angelo Karaboyas, Hirotaka Komaba, Masatomo Taniguchi, Takanobu Nomura, Brian A. Bieber, Patricia De Sequera, Anders Christensson, Ronald L. Pisoni, Bruce M. Robinson, Masafumi Fukagawa

**Affiliations:** 1 Division of Clinical Nephrology and Rheumatology, Niigata University Graduate School of Medical and Dental Sciences, 1-757 Asahimachi-dori, Niigata, 951-8510 Japan; 2 Arbor Research Collaborative for Health, Ann Arbor, MI USA; 3 Division of Nephrology, Endocrinology and Metabolism, Tokai University School of Medicine, Isehara, Japan; 4 Fukuoka Renal Clinic, Fukuoka, Japan; 5 Medical Affairs Department, Kyowa Hakko Kirin Co. Ltd., Tokyo, Japan; 6 University Hospital Infanta Leonor, Madrid, Spain; 7 Department of Nephrology, Skåne University Hospital, Malmö-, Lund, Sweden

**Keywords:** Parathyroid hormone, Hemodialysis, Dialysis outcomes and practice patterns study, PTH slope, Japanese society for Dialysis therapy

## Abstract

**Background:**

High-circulating level of parathyroid hormone (PTH) is associated with elevated mortality in dialysis patients. The Japanese Society for Dialysis Therapy guideline suggests a lower PTH target than other international guidelines; thus, PTH control may differ in Japan compared with other regions, and be associated with mortality.

**Methods:**

We analyzed data from hemodialysis patients with ≥3 measurements of PTH during the first 9 months after enrollment in the Dialysis Outcomes and Practice Patterns Study (DOPPS) phases 4–5 (2009–2015). PTH control was assessed by the mean, slope, and mean squared error (MSE) of all PTH measurements over the 9-month run-in period. Distribution of each PTH control was assessed by regions (Europe/Australia/New Zealand [Eur/ANZ], Japan and North America) and dialysis vintage. Mortality rates were compared across PTH control categories using Cox regression models.

**Results:**

Mean PTH was lower in Japan than in other regions across dialysis vintage categories. In patients with dialysis vintage < 90 days, PTH level was more likely to decline > 5% per month in Japan (48% of patients) versus Eur/ANZ (35%) and North America (35%). In patients with dialysis vintage > 1 year, Japanese patients maintained steady PTH, while patients in Eur/ANZ and North America were more likely to experience a PTH increase. Mean PTH was associated with mortality in the overall samples (highest mortality rate for PTH > 600 pg/mL, hazard ratio, 1.35; 95% confidence interval, 1.20 to 1.52 vs PTH 200–399 pg/mL), and the association was obvious in the prevalent patients (hazard ratio, 1.44; 95% confidence interval, 1.26 to 1.65). PTH slope and MSE did not show significant association with mortality in the overall sample as well as in subjects stratified both by region and dialysis vintage.

**Conclusion:**

PTH control in hemodialysis patients, as measured by keeping a stable PTH level over 9 months, was observed in Japan contrasted with other regions. High PTH mean, but not increased PTH slope and MSE, was associated with mortality especially in prevalent patients.

## Background

Mortality of patients with chronic kidney disease (CKD), especially undergoing maintenance hemodialysis (HD) treatment remain higher than the general population [1]. Abnormal mineral and bone metabolism induced by kidney disease accelerate cardiovascular disease, fracture, and other adverse clinical outcomes [2–4]. High serum parathyroid hormone (PTH) level is associated with higher all cause and cardiovascular mortality as well as higher incidence of fracture in patients with maintenance HD [5–10]. Lower PTH level is associated with mortality [5, 7–9] probably owing to malnutrition, and also associated with adynamic bone disease owing to skeletal resistance to PTH [11]. One of important strategies to improve survival in maintenance dialysis patients will be maintaining PTH levels lower with interventions, such as intravenous (IV) vitamin D analogs and calcimimetics [12].The target level of intact PTH was recommended from 150 pg/mL to 300 pg/mL by the National Kidney Foundation in 2003 [13]. The clinical practice guideline for CKD-mineral and bone disorder (CKD-MBD) from Kidney Disease: Improving Global Outcomes (KDIGO) in 2010 [14] suggests maintaining PTH level in the range of approximately 2 to 9 times the upper normal limit for assay, which is the same with a renewed guideline in 2017 considering better survival [15]. The international Dialysis Outcomes and Practice Patterns Study (DOPPS) reported that serum PTH levels have increased in all regions except for Japan, while prescriptions of IV vitamin D analogs and cinacalcet increased during a 15-year period [5]. On the other hand, the Japanese Society for Dialysis Therapy (JSDT) guideline suggests a low and narrow PTH target (60–240 pg/mL) [16], and the serum PTH level remained low and stable in Japanese HD patients [5]. We can find the difference of absolute level of PTH in each regions while there is little information about the practice for PTH control in Japan compared with other regions.

HD practice, such as vascular access, treatment time and dialysis adequacy, has changed globally; however, mortality in Japanese maintenance HD patients was better than in other regions [17, 18]. The management of PTH in HD patients may explain the difference of survival in Japan and other regions. To understand PTH management in each region, it should be evaluated not only with PTH level at one-point measurement, but with several PTH control during certain periods of dialysis treatment, including mean as cumulative expose with PTH, slope as trend of PTH control, or variability as trend of PTH stability. PTH control, as measured by these 3 characteristics, will be different across regions and dialysis vintage and may be associated with survival in dialysis patients. On the basis of data from the international DOPPS, we investigated measures of PTH control in HD patients in Japan compared with other countries and evaluate their association with mortality.

## Methods

### Patients and data collection

The DOPPS is an international prospective cohort study of patients on in-center HD who are ≥18 years of age, which is currently in phase 6. At each phase, a random sample of chronic HD facilities was selected; within each participating facility, a census of patients on prevalent in-center HD was used to select at random 20–40 patients. Study approval was obtained by a central institutional review board and local ethics committees, as required. Data on monthly laboratory values, medication prescription, and death were abstracted from patient records at baseline and every 4 months. In this study, HD patients with ≥3 measurements of PTH during the first 9 months in DOPPS phase 4 and 5 (2009–2015) were selected in Japan, North America, and Europe / Australia / New Zealand (Eur/ANZ). For the majority of the study period, intact PTH was the only assay available for clinical practice; 10% of DOPPS facilities reported using biointact PTH assays. Patients who had a parathyroidectomy prior to DOPPS enrollment were excluded.

### Exposure and outcome

We first log-transformed PTH due to the skewed distribution. We then used linear regression to model log (PTH) separately for each patient over the 9-month run-in period (3 to 9 PTH measurements), with time (month) as the only covariate. Three different measures of PTH control were defined: PTH slope, PTH-mean squared error (MSE), and PTH mean. The slope of log (PTH) was estimated from the regression models, with PTH slope parameterized as % change (per month) over the 9-month run-in period. The MSE of the model, representing PTH variability along the fitted slope, was calculated as the mean of the squared residuals from the regression models. PTH mean was defined as the geometric mean, exp. (mean [log (PTH)]) of all measurements over the 9-month run-in period to lessen the influence of outlier values.

Distributions of these three measures of PTH control were assessed by regions (Eur/ANZ, North America, and Japan) and dialysis vintage at DOPPS enrollment (< 90 days, 90 days-1 year and > 1 year). We also present prescription of PTH-lowering therapies (active vitamin D and cinacalcet; any usage over the 9 month run-in period) by region and dialysis vintage.

### Statistical analyses

To investigate the association between each of these three measures of PTH control with all-cause mortality, we used Cox regression using robust sandwich covariance estimators to account for facility clustering, and stratified by DOPPS phase, country, and indicators for black race and large dialysis organization (US only). Follow-up began after the 9-month run-in period and continued until death, study phase end, or 7 days after leaving the facility due to loss to follow-up, transplantation, or modality switch (whichever occurred first). Exposures were categorized into five groups to assess the functional form of the associations. Covariate adjustment was made for age, sex, vintage, catheter use, 13 comorbidities (Table 1), and mean values of body mass index (BMI), normalized protein catabolic rate (nPCR), hemoglobin (Hgb), creatinine, and albumin over the 9-month run-in period. Other covariates that may potentially be mediators on the causal pathway between PTH control and mortality were additionally adjusted for in a sensitivity analysis, but were not included in the primary analysis: prescription of phosphate binder, cinacalcet, IV and oral vitamin D, plus the mean and slope of serum phosphorus and calcium calculated over the 9-month run-in period (as done for PTH above).

**Table 1:** Patient characteristics by region

Patient characteristic	Europe	Japan	North America
N patients	5910	2627	18,251
Characteristics at study entry			
Age (years)	65.9 ± 14.7	64.3 ± 12.3	62.7 ± 14.9
Sex (% male)	62%	66%	55%
Race (% black)	2%	0%	37%
Vintage (years)	1.9 (0.4, 5.1)	3.8 (0.7, 9.2)	2.0 (0.5, 4.6)
Catheter use (%)	26%	1%	32%
Dialysate calcium (mEq/L)	2.9 ± 0.3	2.8 ± 0.2	2.5 ± 0.2
Mean over 9-month run-in period			
BMI (kg/m^2^)	26.3 ± 5.5	21.5 ± 3.5	28.7 ± 7.1
Normalized PCR (g/kg/day)	1.00 ± 0.22	0.93 ± 0.18	0.95 ± 0.22
Hgb (g/dL)	11.5 ± 1.0	10.6 ± 0.9	11.0 ± 0.9
Serum creatinine (mg/dL)	7.9 ± 2.4	10.4 ± 2.8	7.9 ± 2.8
Serum albumin (g/dL)	3.73 ± 0.43	3.71 ± 0.36	3.76 ± 0.38
Serum calcium (mg/dL)	9.0 ± 0.6	8.9 ± 0.6	9.0 ± 0.6
Serum phosphorus (mg/dL)	4.9 ± 1.2	5.4 ± 1.0	5.2 ± 1.2
PTH (pg/mL)*	233 (140, 377)	126 (77, 192)	283 (194, 425)
Slope over 9-month run-in period			
Calcium (mg/dL per year)	0.1 (−0.5, 0.8)	0.1 (− 0.4, 0.7)	0.1 (− 0.5, 0.7)
Phosphorus (mg/dL per year)	−0.1 (− 1.5, 1.4)	−0.2 (− 1.5, 1.2)	0.1 (− 1.3, 1.5)
PTH (% change per month)	0.6 (− 6.1, 7.1)	−1.0 (− 7.0, 4.9)	0.5 (− 6.0, 7.1)
MSE over 9-month run-in period**			
Calcium	0.1 (0.1, 0.2)	0.1 (0.1, 0.2)	0.1 (0.1, 0.2)
Phosphorus	0.6 (0.3, 1.1)	0.5 (0.3, 1.0)	0.7 (0.4, 1.3)
Log(PTH)	0.1 (0.0, 0.2)	0.1 (0.0, 0.2)	0.1 (0.0, 0.3)
Medications over 9-month run-in period (% any prescription over 9 months)			
Phosphate binder	85%	88%	80%
Cinacalcet	23%	22%	22%
IV vitamin D	24%	45%	71%
Oral vitamin D	51%	45%	26%
Any active vitamin D	69%	81%	86%
Comorbid conditions (%)			
Coronary artery disease	37%	28%	34%
Congestive heart failure	20%	19%	30%
Cerebrovascular disease	16%	13%	11%
Peripheral vascular disease	31%	15%	15%
Other cardiovascular disease	31%	24%	19%
Hypertension	87%	81%	82%
Diabetes	38%	41%	62%
Neurologic disease	12%	7%	8%
Psychiatric disorder	18%	5%	15%
Lung disease	15%	4%	12%
Cancer (non-skin)	16%	10%	7%
Gastrointestinal bleeding	5%	4%	3%
Recurrent cellulitis, gangrene	9%	3%	9%

Mean ± SD or median (IQR) or % shown

*Geometric mean: Exp(mean(log(PTH)))

**MSE mean squared error from fitted regression model over 9-month run-in period

Effect heterogeneity by dialysis vintage was assessed because PTH slopes and means may differ by vintage; PTH levels among patients on dialysis for many years may reflect treatment, while PTH levels among incident dialysis patients may depend more on predialysis PTH levels. Effect heterogeneity by region was assessed because PTH targets vary greatly by region; the same PTH level (e.g., 300 pg/mL) may be in-target in one region (US), but out of target in another region (Japan), and thus likely treated differently.

To deal with missing covariate data, we used multiple imputation, assuming data were missing at random. Missing covariate values were multiply imputed using the Sequential Regression Multiple Imputation Method by IVEware [19]. Results from 20 such imputed data sets were combined for the final analysis using Rubin’s formula [20]. The proportion of missing data was below 10% for all variables used for covariate adjustment, with the exception of eight comorbidities (coronary artery disease, cerebrovascular disease, other cardiovascular disease, neurologic disease, psychiatric disorder, lung disease, gastrointestinal bleeding, and recurrent cellulitis/gangrene; 40% missing) that were not collected in a subset of US facilities. All analyses were conducted using SAS software, version 9.4 (SAS institute, Cary, NC).

## Results

### Comparing PTH control practices in Japan with Europe and North America

**Fig. 1:**
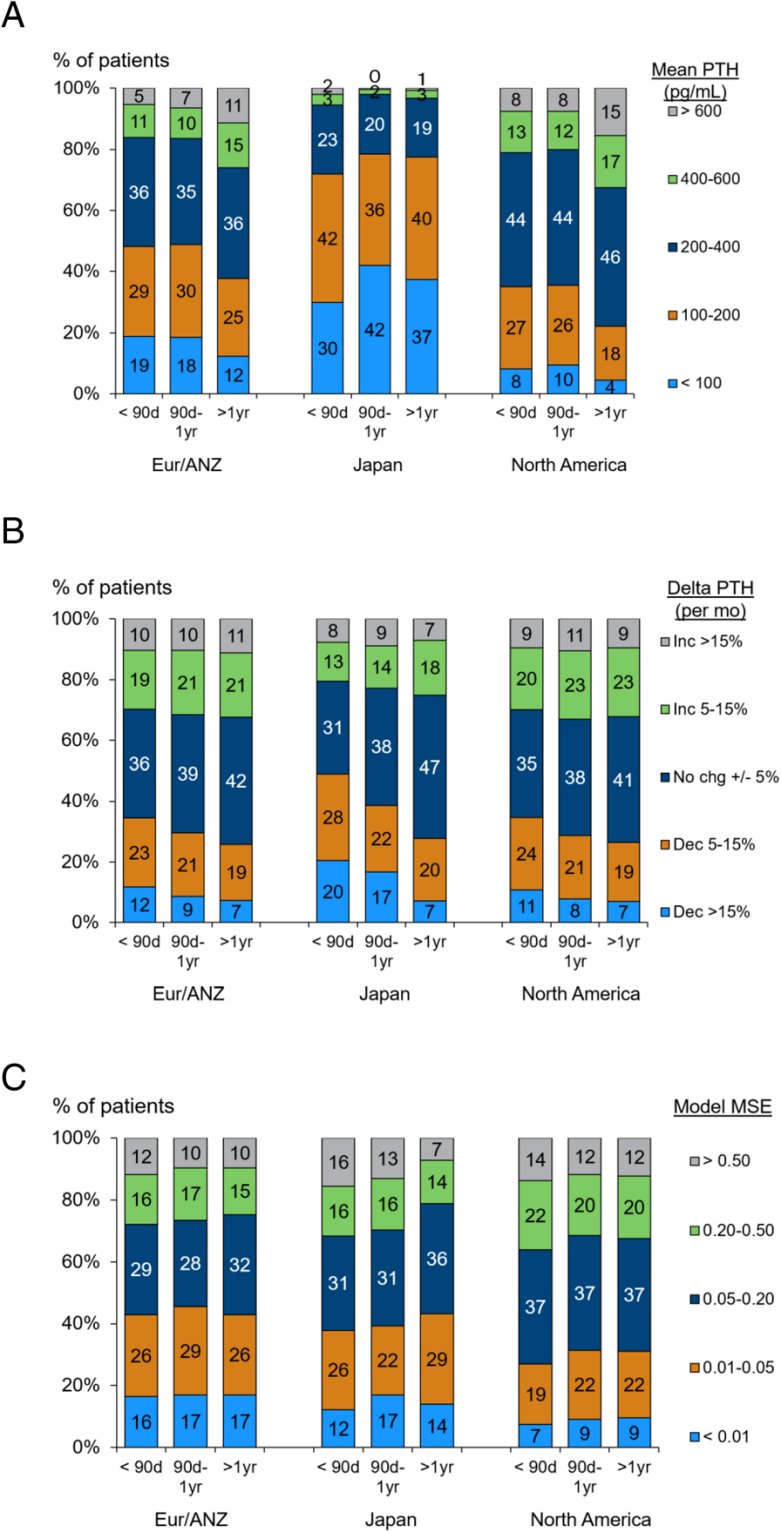
Three measures of PTH control, by region and dialysis vintage. PTH control was defined as: (**a**) mean of absolute PTH (PTH mean); (**b**) slope of log (PTH) (PTH slope); and (**c**) mean squared error of PTH (PTH-MSE) over the 9 month run-in period. Each figure shows the distribution of PTH control by region [Europe/Australia/New Zealand (Eur/ANZ), North America and Japan] and dialysis vintage at DOPPS enrollment (< 90 days, 90 days-1 year and > 1 year)

Our sample included 5910 patients in Eur/ANZ, 2627 in Japan, and 18,251 in North America. Table 1 shows patient characteristics in those regions. In Japan, patients showed longer dialysis vintage, less use of catheter as vascular access, and lower BMI. The median (IQR) of patients’ geometric mean PTH over 9 months was lower in Japan, as compared with Eur/ANZ and North America [Japan: 126 (77, 192) pg/mL, Eur/ANZ: 233 (140, 377) and North America: 283 (194, 425) pg/mL]. PTH slope was centered near zero (no change) in all three regions, as each region included many patients with both increasing, decreasing, and steady PTH: the median (interquartile range [IQR]) % change in PTH per month was − 1.0 (− 7.0, 4.9) in Japan, 0.6 (− 6.1, 7.1) in Eur/ANZ, and 0.5 (− 6.0, 7.1) in North America. Next, we assessed the 3 measures of PTH control, including mean of PTH, slope, and MSE by region and dialysis vintage (Fig. 1). Geometric mean of PTH over 9 months was much lower in Japan than in other regions across dialysis vintage categories (Fig. 1a). In Japan, PTH mean showed highest in patients with dialysis vintage < 90 days (median 139 pg/mL; IQR: 89–217), and was getting smaller with vintage (Fig. 1a). Among patients with baseline vintage < 90 days, the median (IQR) PTH mean was 206 (122–316) in Eur/ANZ and 249 (169–372) in North America; in contrast to Japan, PTH mean was larger at greater dialysis vintage in these regions (Fig. 1a). In patients with dialysis vintage < 90 days, PTH level was more likely to decline > 5% per month in Japan (48% of patients) versus Eur/ANZ (35%) and North America (35%) (Fig. 1b). In patients with dialysis vintage > 1 year, Japanese patients were most likely to maintain steady PTH (PTH slope within +/− 5% per month: 47% in Japan vs. 42% in Eur/ANZ and 41% in North America), and patients in Eur/ANZ and North America more likely to experience increase in PTH (Fig. 1b). When we assessed the distribution of within-patient residuals of log (PTH) in Japan, higher PTH-MSE was found in patients with dialysis vintage < 90 days, while MSE got smaller in those with dialysis vintage > 90 days, an indicator of more stable PTH control (Fig. 1c). There was no difference of residuals of log (PTH) with dialysis vintage both in Eur/ANZ and North America (Fig. 1c).

**Fig. 2:**
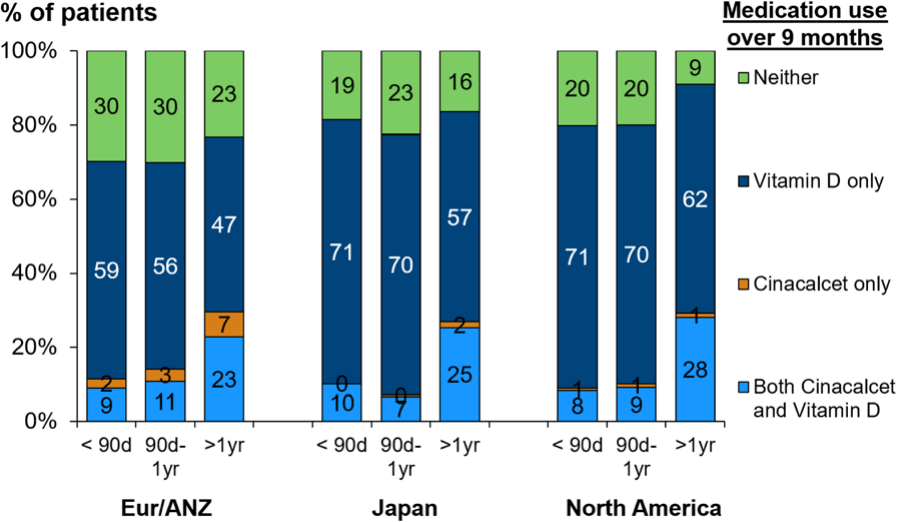
Prescription of PTH-lowering therapies by region and dialysis vintage. The figure shows any prescription of active vitamin D or cinacalcet over the subsequent 9 month run-in period, by region [Europe/Australia/New Zealand (Eur/ANZ), North America and Japan] and dialysis vintage at DOPPS enrollment (< 90 days, 90 days-1 year and > 1 year)

The prescription of PTH-lowering therapies (any over the 9 month run-in period) is illustrated in Fig. 2 by region and dialysis vintage. Cinacalcet use was similar across regions: about 10% during the first year of HD and 30% among patients on HD for over 1 year. Active vitamin D use also increased with dialysis vintage, and was lower in Eur/ANZ (where oral vitamin D is preferred over IV vitamin D) than in North American and Japan, consistent with a previous DOPPS report [5].

### Association between PTH control practices and mortality

Median follow-up was 14.4 (IQR, 6.8–23.7) months, and associations between the 3 measures of PTH control and mortality are shown in Table 3, with Model 3 being the primary analysis. PTH slope had minimal association with mortality (Table 2, Model 3). Results further stratified by region are shown in Table 3, and results stratified by vintage category are shown in Table 4. We also observed minimal association between PTH-MSE and mortality in the overall sample (Table 2, Model 3) as well as in subjects stratified both by region (Table 3) and dialysis vintage (Table 4). Regarding the geometric mean of PTH over 9 months, patients with the highest mean PTH (> 600 pg/mL) had the highest mortality rate in the adjusted model (hazard ratio [HR] = 1.35 (95% confidence interval [CI] 1.20–1.52) vs. PTH 200–399 pg/mL) (Table 2, Model 3). The association weakened after adjusting with several covariates related with calcium and phosphate control (Table 2, HR 1.19 [95% CI 1.04–1.35] in Model 3a), which may reflect an over-adjustment for factors in the causal pathway. The effect was much clearer in prevalent patients who had been on dialysis for over 1 year at study enrollment (vintage > 1 year) [HR = 1.44 (95% CI 1.26–1.65)], as compared with those with dialysis vintage < 90 days [HR = 1.13 (95% CI 0.75–1.77)] and 90 days-1 year [HR = 1.00 (95% CI 0.74–1.36)] (Table 4).

**Table 2:** Association between PTH slope/residuals/mean and mortality, by level of adjustment

Exposure	N (%)	Model 1	Model 2	Model 3	Model 3a
PTH slope (% change per month)					
Decreased > 15%	2189 (8%)	1.09 (0.97–1.22)	1.08 (0.96–1.21)	1.10 (0.98–1.25)	1.13 (1.00–1.28)
Decreased 5–15%	5479 (20%)	1.12 (1.03–1.21)	1.05 (0.97–1.14)	1.06 (0.98–1.15)	1.07 (0.98–1.16)
No change ±5%	10,776 (40%)	1 (Ref.)	1 (Ref.)	1 (Ref.)	1 (Ref.)
Increased 5–15%	5750 (21%)	1.05 (0.97–1.13)	1.04 (0.97–1.13)	1.05 (0.97–1.13)	1.03 (0.96–1.12)
Increased > 15%	2594 (10%)	1.07 (0.96–1.19)	1.06 (0.95–1.18)	1.08 (0.97–1.20)	1.04 (0.93–1.16)
PTH mean squared residuals					
< 0.01	2967 (11%)	0.98 (0.89–1.09)	1.01 (0.91–1.13)	1.01 (0.90–1.12)	1.01 (0.90–1.13)
0.01–0.05	6205 (23%)	0.93 (0.86–1.01)	0.94 (0.87–1.02)	0.93 (0.86–1.01)	0.94 (0.86–1.02)
0.05–0.20	9481 (35%)	1 (Ref.)	1 (Ref.)	1 (Ref.)	1 (Ref.)
0.20–0.50	5043 (19%)	1.02 (0.94–1.11)	0.99 (0.91–1.08)	1.00 (0.92–1.09)	1.00 (0.92–1.09)
> 0.50	3092 (12%)	0.95 (0.86–1.05)	0.97 (0.88–1.08)	0.98 (0.89–1.09)	0.99 (0.89–1.11)
PTH geometric mean (pg/mL)					
< 100	2975 (11%)	1.28 (1.16–1.41)	1.01 (0.91–1.12)	1.00 (0.90–1.11)	0.99 (0.89–1.11)
100–199	6395 (24%)	1.09 (1.01–1.18)	0.93 (0.86–1.00)	0.92 (0.85–1.00)	0.94 (0.87–1.02)
200–399	10,887 (41%)	1 (Ref.)	1 (Ref.)	1 (Ref.)	1 (Ref.)
400–599	3666 (14%)	0.92 (0.83–1.01)	1.10 (1.00–1.22)	1.11 (1.01–1.22)	1.05 (0.95–1.17)
≥ 600	2865 (11%)	0.90 (0.81–1.00)	1.33 (1.18–1.50)	1.35 (1.20–1.52)	1.19 (1.04–1.35)

HR (95% CI) of all-cause mortality for each exposure displayed

Model 1: Stratified by phase, country, US-black race

Model 2: Model 1 + adjusted for age, sex, vintage, catheter use, 13 comorbidities, mean values of BMI, nPCR, Hgb, creatinine, albumin over 9-month run-in period

Model 3: Model 2 + simultaneous adjustment for all 3 exposures (PTH slope/residuals/mean)

Model 3a: Model 3 + adjusted for potential mediators: prescription of phosphate binders, cinacalcet, IV vitamin D, and oral vitamin D, P mean, (P mean)^2, P slope, Ca mean, and Ca slope over 9-month run-in period

**Table 3:** Association between PTH slope/residuals/mean and mortality, by region

Exposure	Europe + Australia/NZ		Japan		North America	
	N (%)	Adjusted* HR (95% CI)	N (%)	Adjusted* HR (95% CI)	N (%)	Adjusted* HR (95% CI)
PTH slope (% change per month)						
Decreased > 15%	487 (8%)	1.08 (0.85–1.37)	271 (10%)	1.14 (0.72–1.80)	1431 (8%)	1.12 (0.97–1.30)
Decreased 5–15%	1166 (20%)	0.92 (0.77–1.09)	566 (22%)	1.25 (0.92–1.71)	3747 (21%)	1.10 (1.00–1.21)
No change ±5%	2375 (40%)	1 (Ref.)	1152 (44%)	1 (Ref.)	7249 (40%)	1 (Ref.)
Increased 5–15%	1244 (21%)	1.05 (0.91–1.21)	443 (17%)	0.81 (0.56–1.18)	4063 (22%)	1.07 (0.98–1.18)
Increased > 15%	638 (11%)	0.94 (0.75–1.17)	195 (7%)	1.57 (0.95–2.59)	1761 (10%)	1.11 (0.98–1.26)
PTH mean squared residuals						
< 0.01	991 (17%)	1.09 (0.92–1.30)	373 (14%)	1.13 (0.75–1.72)	1603 (9%)	0.94 (0.81–1.10)
0.01–0.05	1576 (27%)	0.95 (0.82–1.10)	728 (28%)	1.09 (0.78–1.53)	3901 (21%)	0.92 (0.83–1.02)
0.05–0.20	1833 (31%)	1 (Ref.)	901 (34%)	1 (Ref.)	6747 (37%)	1 (Ref.)
0.20–0.50	920 (16%)	0.91 (0.76–1.09)	384 (15%)	1.17 (0.74–1.88)	3739 (20%)	1.00 (0.91–1.11)
> 0.50	590 (10%)	1.04 (0.83–1.31)	241 (9%)	1.22 (0.73–2.05)	2261 (12%)	0.94 (0.83–1.06)
PTH geometric mean (pg/mL)						
< 100	862 (15%)	1.04 (0.86–1.25)	983 (37%)	1.22 (0.83–1.79)	1130 (6%)	0.97 (0.83–1.13)
100–199	1598 (27%)	0.90 (0.76–1.05)	1041 (40%)	1.35 (0.93–1.97)	3756 (21%)	0.93 (0.84–1.02)
200–399	2120 (36%)	1 (Ref.)	514 (20%)	1 (Ref.)	8253 (45%)	1 (Ref.)
400–599	774 (13%)	1.23 (1.01–1.51)	65 (2%)	–	2827 (15%)	1.04 (0.93–1.17)
≥ 600	556 (9%)	1.26 (0.97–1.62)	24 (1%)	–	2285 (13%)	1.31 (1.14–1.50)

*Adjusted as in Table 2, Model 3

**Table 4:** Association between PTH slope/residuals/mean and mortality, by vintage

Exposure	Vintage < 90 days		Vintage 90 days - 1 year		Vintage > 1 year	
	N (%)	Adjusted* HR (95% CI)	N (%)	Adjusted* HR (95% CI)	N (%)	Adjusted* HR (95% CI)
PTH slope (% change per month)						
Decreased > 15%	459 (12%)	1.06 (0.74–1.52)	445 (9%)	1.12 (0.83–1.52)	1189 (7%)	1.16 (1.00–1.35)
Decreased 5–15%	935 (24%)	1.06 (0.81–1.38)	1075 (21%)	0.92 (0.75–1.14)	3267 (19%)	1.13 (1.03–1.24)
No change ±5%	1373 (35%)	1 (Ref.)	1949 (38%)	1 (Ref.)	7082 (42%)	1 (Ref.)
Increased 5–15%	766 (20%)	1.20 (0.91–1.58)	1095 (22%)	0.94 (0.77–1.14)	3689 (22%)	1.06 (0.97–1.17)
Increased > 15%	374 (10%)	1.34 (0.99–1.82)	527 (10%)	0.79 (0.60–1.06)	1612 (10%)	1.15 (1.01–1.31)
PTH mean squared residuals						
< 0.01	386 (10%)	1.09 (0.79–1.51)	589 (12%)	1.23 (0.96–1.56)	1936 (12%)	0.98 (0.86–1.12)
0.01–0.05	844 (22%)	0.78 (0.59–1.03)	1214 (24%)	0.97 (0.81–1.16)	3964 (24%)	0.96 (0.87–1.06)
0.05–0.20	1358 (35%)	1 (Ref.)	1757 (35%)	1 (Ref.)	5981 (36%)	1 (Ref.)
0.20–0.50	793 (20%)	0.83 (0.64–1.08)	950 (19%)	0.91 (0.74–1.11)	3081 (18%)	1.07 (0.97–1.19)
> 0.50	526 (13%)	0.77 (0.57–1.05)	581 (11%)	0.88 (0.66–1.17)	1877 (11%)	1.05 (0.92–1.19)
PTH geometric mean (pg/mL)						
< 100	482 (12%)	1.10 (0.81–1.49)	739 (15%)	0.93 (0.75–1.16)	1658 (10%)	1.07 (0.94–1.22)
100–199	1114 (29%)	0.92 (0.74–1.15)	1421 (28%)	0.93 (0.78–1.10)	3663 (22%)	0.96 (0.87–1.05)
200–399	1581 (40%)	1 (Ref.)	2034 (40%)	1 (Ref.)	6838 (41%)	1 (Ref.)
400–599	470 (12%)	0.82 (0.56–1.20)	554 (11%)	1.15 (0.89–1.49)	2511 (15%)	1.13 (1.01–1.26)
≥ 600	260 (7%)	1.13 (0.73–1.77)	343 (7%)	1.00 (0.74–1.36)	2169 (13%)	1.44 (1.26–1.65)

*Adjusted as in Table 2, Model

## Discussion

In this study, we reported three different measures of PTH control in HD patients from three regions using worldwide DOPPS data. We compared PTH control in Japan versus other countries and evaluated the association between PTH control and mortality. In Japan, we found that PTH level decreased over the first year of dialysis (over 9 months among patients on dialysis < 90 days at DOPPS enrollment), and remained stable thereafter. In contrast, PTH level tended to increase with dialysis vintage in both Eur/ANZ and North America. In the parameters of PTH control, when analyzing PTH control and mortality, the strongest association was observed for a high PTH mean (> 600 pg/mL) sustained over the 9 months, especially among prevalent (vintage > 1 year) patients.

Practice patterns for dialysis treatment are different in each country, and survival in Japan is superior to that in other regions [17, 18]. Several factors, including race, purity of dialysate, and variation in vascular access are thought to be associated with mortality [18]. Previous reports showed that high level of absolute PTH is one of risk of mortality in HD patients, and the level in Japan is smaller than that in other regions [5]. We then hypothesized that the practice pattern for PTH control is different between Japan and other regions, and that measures of PTH control are associated with mortality in HD patients.

In this study, PTH slope in Japan was lower in dialysis vintage < 90 days, kept low and stable after 1 year of dialysis duration. JSDT guideline for CKD-MBD management recommends PTH target from 60 to 240 pg/mL for better survival [16]. In the guideline, frequency of PTH measurement is recommended once every 3 months, however, monthly measurement of PTH is required until the PTH concentration is stabilized in target range with intervention [16]. JSDT guideline was recommended to manage PTH from 60 to 180 pg/mL in 2008 [21] which was the narrow target to be achieved, and revised it to PTH 60–240 pg/mL with reanalyzed JSDT data and for avoidance of very low PTH level. Furthermore, adequate PTH levels makes easy to control serum phosphorus and calcium [22]. Adopting JSDT guideline, the physicians in Japan may try to reach the target aggressively soon after initiation of dialysis treatment. Since prescription rate of PTH-lowering therapies in Japan was not high compared with other regions (Fig. 2), it is possible that effectiveness of medication may be different between Japan and other regions. Another possibility of decreasing PTH may be that initiation of dialysis treatment induces reduction of PTH due to supplementation of calcium from dialysate. In addition to aggressive intervention for PTH control after initiation of dialysis, proper PTH control at the pre-dialysis stage will be important to get better PTH control in incident dialysis patients while a European group reported insufficient CKD-MBD management in non-dialysis patients even with regular nephrology care [23]. On the other hand, Eur/ANZ and North America showed higher PTH level with dialysis vintage, contrary to the trend in Japan. One major reason for the difference of PTH level between Japan and other regions may be due to the CKD-MBD guidelines that physicians adopt. The revised KDIGO guideline in 2017 recommends target of PTH level as 2 to 9 times the upper limit of the assay [15]. Physicians in Eur/ANZ and North America who adopt the KDIGO guideline will try to control PTH level not exceeding 600 pg/mL, which is about 9 times the upper limit of the assay; however, 11% and 16% of dialysis patients in Eur/ANZ and North America, respectively, have serum PTH level over 600 pg/mL. In this study, there was not so much difference of MBD treatment between Japan and other regions [ex. Medication rate of cinacalcet: 23% vs. 22% vs. 23% and any active vitamin D: 69% vs. 81% vs. 86% in Eur/ANZ vs. Japan vs. North America, respectively, Table 1 and Fig. 2]. Then, the intervention not only in predialysis stage, but in earlier dialysis vintage and/or before reaching high PTH level may be recommended to avoid excessive increase of PTH.

There are several reports about the association between absolute PTH level and mortality in dialysis patients [5–9]. In this study, we examined the relation of parameters for PTH control and survival using worldwide DOPPS data, and found that high PTH mean for 9-month periods was associated with mortality, but not high PTH slope, or a large MSE that reflects greater variation around the fitted slope. The association between mean PTH > 600 pg/mL and mortality was strong in the primary model and even remained after adjustment for potential mediators related to calcium and phosphate control (Table 2, Model 3 vs. Model 3a). PTH mean > 600 pg/mL increased mortality risk, and the effect was strongest in patients with dialysis vintage > 1 year as compared with those with dialysis vintage < 90 days and 90 days-1 year (Table 4). In incident patients, the association between PTH and mortality was not strong, potentially because PTH will be changed easily with control of calcium and phosphorus. The stronger association in prevalent patients may be owing to sustainable hyperparathyroidism with parathyroid hyperplasia which may induce vascular calcification and abnormal bone metabolism with the prolonged exposure of PTH to vascular and bone. Fibroblast growth factor 23 (FGF23) may be associated with clinical outcomes in patients with high PTH mean because high PTH is related with higher FGF23 level which is strongly associated with mortality in CKD and dialysis patients [24, 25]. In this study, data of FGF23 were not widely available, and a future study will be needed to examine FGF23 control in dialysis patients to understand better CKD-MBD management. Although increased PTH slope did not show increase of risk for mortality, a steep (> 15% per month) decrease in PTH was associated with a slightly elevated – not lower – mortality rate, which was strongest in patients with dialysis vintage more than 1 year (Table 4). The Current Management of Secondary Hyperparathyroidism, a Multi-Center Observational Study (COSMOS) study showed the survival benefit with increase of PTH level in HD patients with baseline PTH < 168 pg/mL, but not those with decrease of PTH [8]. Another study showed that a small increase of PTH < 300 pg/mL within 12 months was associated with better survival, while decreased PTH showed the increase of mortality in prevalent HD patients whose baseline PTH was 205 (116.5, 400) pg/mL [26]. One of the reasons why decrease of PTH is associated with increase of mortality may be malnutrition [27]. In fact, our data showed that HR of mortality in patients with PTH mean < 100 pg/mL decreased when several markers associated with nutrition were added as covariates (Table 2, Model 1 vs. Model 2). Thus, to keep stable and lower PTH level will lead to better survival in prevalent HD patients. Japanese practice patterns for PTH management, such as decreased PTH levels after initiation of dialysis treatment, and keeping stable and lower PTH levels in the maintenance dialysis phase, may be better to avoid PTH > 600 pg/mL, as well as major increases or decreases of PTH in prevalent patients. To clarify the usefulness of Japanese PTH management, further studies will be needed to examine survival benefit with stable and low PTH management across countries.

The DOPPS study design allowed us to present trends in PTH control and the association with outcomes in a cohort of real-world patients, similar to what physicians may encounter when rounding in the dialysis unit. However, we must acknowledge some important limitations. Multiple assays are available for measurement of intact PTH, and large interassay variability may have contributed to misclassification of specific patients. Assuming this variation is random with respect to patient characteristics, this would tend to bias associations of PTH, with mortality to the null. As with any observational study, the reported associations do not prove causality and may be affected by unmeasured confounders. HD patients with ≥3 measurements of PTH during the first 9 months were enrolled. This would tend to bias associations that may select dialysis units to control PTH management carefully, or patients who have to measure PTH frequently. Patients on HD were enrolled in this study, and there was no information regarding CKD-MBD treatment in predialysis stage.

## Conclusion

PTH control, as measured by keeping a stable PTH level over 9 months, was better in Japan versus other regions. High PTH mean, but not increased PTH slope and MSE, was associated with mortality, especially among prevalent hemodialysis patients. PTH management of treating aggressively after initiation of dialysis, and subsequently sustaining PTH within a low target range, might be preferable to avoid the potentially harmful consequences of high PTH level in HD patients.

## Abbreviations

BMI: Body mass index
CI: Confidence interval
CKD: Chronic kidney disease
CKD-MBD: CKD-mineral and bone disorder
COSMOS: Current Management of Secondary Hyperparathyroidism, a Multi-Center Observational Study
DOPPS: Dialysis Outcomes and Practice Patterns Study
Eur/ANZ: Europe / Australia / New Zealand
HD: Hemodialysis
Hgb: Hemoglobin
HR: Hazard ratio
IQR: Interquartile range
IV: Intravenous
JSDT: Japanese Society for Dialysis Therapy
KDIGO: Kidney Disease: Improving Global Outcomes
MSE: Mean squared error
nPCR: Normalized protein catabolic rate
PTH: Parathyroid hormone
